# Preference for Solitude and Mobile Phone Addiction Among Chinese College Students: The Mediating Role of Psychological Distress and Moderating Role of Mindfulness

**DOI:** 10.3389/fpsyg.2021.750511

**Published:** 2021-12-17

**Authors:** Wan-Yi Chen, Lei Yan, Yi-Ren Yuan, Xiao-Wei Zhu, Yan-Hong Zhang, Shuai-Lei Lian

**Affiliations:** College of Education and Sports Science, Yangtze University, Jingzhou, China

**Keywords:** preference for solitude, psychological distress, mobile phone addiction, mindfulness, collectivistic culture

## Abstract

**Background:** With the increasing incidence of mobile phone addiction, the potential risk factors of mobile phone addiction have attracted more and more researchers’ attention. Although various personality trait factors have been proven to be significant predictors of mobile phone addiction, limited attention has been paid to preference for solitude. Considering the adverse impacts of preference for solitude in the context of collectivistic societies and its possible negative effect on mobile phone addiction, this study was designed to examine the relationship between preference for solitude and mobile phone addiction, and to test the mediating role of psychological distress and the moderating role of mindfulness in this relationship.

**Methods:** Data were collected through convenience sampling from a comprehensive university in China. A total of 927 Chinese college students (371 males and 556 females), aged from 16 to 24 (*M*_age_ = 19.89 years, *SD* = 1.22), participated in this study. Their preference for solitude, psychological distress, mindfulness, and mobile phone addiction were measured using well-validated self-report questionnaires.

**Results:** Correlational analyses, sobel test, SPSS macro PROCESS (Model 8) and simple slopes analyses were used for major data analysis. Results showed that preference for solitude was significantly and positively associated with mobile phone addiction, and this link could be mediated by psychological distress. Moreover, the indirect effect of psychological distress in this link was moderated by mindfulness, with this effect being stronger for college students with lower levels of mindfulness. However, mindfulness can not moderate the direct relation between preference for solitude and mobile phone addiction.

**Conclusion:** The present study broadened our knowledge of how and when (or for whom) preference for solitude is related to mobile phone addiction. Education professionals and parents should pay special attention to the psychological distress and mobile phone addiction of college students with high levels of preference for solitude, particularly for those with lower levels of mindfulness.

## Introduction

With the rapid development of mobile information technology today, mobile phones have already become an indispensable part of people’s daily life. According to the 47th China Statistical Report on Internet Development, the number of Internet users in China had increased to 989 million by December 2020, among which the mobile phones subscribers had accounted for 99.7% (China Internet Network Information Center, 2021). Mobile phones can realize multiple functions including entertainment, online shopping, socializing, and monetary gain, which greatly improves the convenience of people’s personal life. Unfortunately, the convenience of mobile phones also brings a series of problems, among which mobile phone addiction should not be overlooked (Yang X. J. et al., 2020). Mobile phone addiction, also known as mobile phone dependence or problematic mobile phone use (Liu et al., 2017a), refers to obvious physical, psychological and social functioning maladjustment in individuals due to the compulsive and excessive use of mobile phones (Cho et al., 2017; Liu et al., 2017b). Mobile phone addiction has become a worldwide problem and received growing research attention over recent years (Lepp et al., 2014; Yang X. et al., 2020). A large number of studies have revealed that mobile phone addiction was closely associated with series of internalizing and externalizing difficulties, such as negative emotions (Elhai et al., 2017), low well-being (Horwood and Anglim, 2019), low self-esteem (Romero-Rodríguez et al., 2020), interpersonal difficulties (Dwyer et al., 2018), and poor sleep quality (Liu et al., 2017b). Furthermore, there is growing evidence identifying that the group of college students are not only the main mobile phones users among young people but also at high risk of mobile phone addiction (Long et al., 2016). Research indicated that the prevalence rate of mobile phone addiction among Chinese undergraduates approximately reached 21.3% (Long et al., 2016), and the potential addiction rate was as high as 58.33% (Chen et al., 2016). Numerous studies have shown that mobile phone addiction has become an important trigger for physical and psychological maladjustment among college students (Lepp et al., 2014; Yang X. et al., 2020). For instance, researchers have found that college students addicted to mobile phones may experience a low level of life satisfaction (Samaha and Hawi, 2016) and college academic failures (Lepp et al., 2014). It’s also noteworthy that the university stage is an important period for the physical and psychological development of individuals (Long et al., 2016). Therefore, it is of great practical significance to identify the potential psychological risk factors of mobile phone addiction among college students and their internal mechanisms.

Personality traits have been proved to be one of the significant predictors of mobile phone addiction (Wang et al., 2019; Zhu et al., 2019; Yang X. et al., 2020). Among various personality factors, preference for solitude has gained researchers’ increasing attention in recent years (Ooi et al., 2018; Zhang et al., 2018; Morneau-Vaillancourt et al., 2021). Preference for solitude, also known as unsociability or social disinterest (Goossens, 2013), refers to the extent to which an individual prefers to be alone (Burger, 1995). Individuals with a high preference for solitude are thought to have low motivation for both social approach and social avoidance (Wang et al., 2013b), and usually show a non-fearful preference for solitary activities (i.e., they refrain from social intercourse, but are not afraid of other people) (Rubin et al., 2009; Coplan and Weeks, 2010). Previous studies on the relationship between preference for solitude and individuals’ psychological and behavioral adaptation have been mostly restricted to western individualistic contexts (Wang, 2016; Coplan et al., 2019) and revealed that solitude has a positive effect on individuals’ development (Long and Averill, 2003). However, preference for solitude has been endowed with different meanings and values in different societies and cultures (Ding et al., 2015; Liu et al., 2015; Coplan et al., 2019).

Western individualistic or self-oriented cultures emphasize autonomy, independence, and the achievement of personal goals (Chen et al., 2006; Coplan et al., 2019). In this context, preference for solitude can be considered as an expression of personal choice, and people may view solitude more positively (Chen and French, 2008). Early Western psychological research has demonstrated that solitude has positive significance for human beings’ psychological adaptation (Larson, 1997; Long and Averill, 2003). For instance, previous studies have found that high preference for solitude not only had a positive after effect on emotional state (Larson, 1997) but also can improve human beings’ well-being (Burger, 1995). Most recently, researchers have also found that high preference for solitude could lead to relaxation as well as reduction in stress (Nguyen et al., 2017). Furthermore, Coplan et al. (2019) integrated research on preference for solitude in the context of Western cultures, and further proposed a speculative theoretical model of the developmental timing effects for preference for solitude, which postulates non-linear variations in the implications of high preference for solitude in the process of individual development. Specifically, the relationship between preference for solitude and social-emotional difficulties would be strengthened over the course of early childhood through to early adolescence, and be weakened from early adolescence to emerging adulthood (Coplan et al., 2019). This implies that in the context of Western individualistic societies, preference for solitude may have a positive effect on college students in later adolescence.

In contrast, collectivistic or group-oriented societies emphasize group interdependence and group harmony, and encourage the maintenance of group well-being over individual interests (Chen and French, 2008; Wang et al., 2013a). Therefore, preference for solitude has been regarded as inconsistent with the social mainstream values, or even as a representation of problematic behavior (Ding et al., 2015; Liu et al., 2015). Studies from Chinese cultural background characterized by collectivism support these views indicating that preference for solitude may have particularly negative effects on individuals’ development (Ding et al., 2018). Specifically, people with high preference for solitude are more likely to experience psychological, social as well as school maladjustment (Liu et al., 2014, 2015). For instance, Wang (2016) showed that preference for solitude was positively associated with negative affect. A longitudinal study also indicated that high levels of preference for solitude could predict peer problems and school difficulties across the school years (Liu et al., 2014).

Preference for solitude may also be an important inducement for individuals’ mobile phone addiction in the era of mobile Internet. First, prior studies have shown that people with high preference for solitude not only experience more loneliness (Liu et al., 2015) but also have lower self-esteem (Wang, 2016). Loneliness and low self-esteem have been proved to be positively associated with problematic mobile phone use (Wang P. et al., 2017; Shen and Wang, 2019). Therefore, individuals with high preference for solitude may have a high risk of mobile phone addiction owing to their loneliness and low levels of self-esteem induced by unsociability. Second, interpersonal adjustment problems associated with preference for solitude may also be a risk factor of individuals’ mobile phone addiction. In the context of group-oriented societies, people who intentionally disengage from the group may be perceived as selfish, “anti-collective” or even deviant, and would be more likely to be isolated by peers and society in the long run (Chen and French, 2008; Liu et al., 2015). Consistent with this view, empirical studies found that individuals with high preference for solitude may have more interpersonal difficulties such as peer avoidance and peer rejection (Liu et al., 2015; Ding et al., 2018). Previous studies have also shown that individuals’ basic psychological needs (e.g., the need to belong) cannot be satisfied when they suffer from interpersonal adjustment difficulties (Xu et al., 2021). According to the theory of Uses and Gratification (Parker and Plank, 2000) and prior studies, people will use mobile phones to satisfy their psychological needs, which may in turn lead to mobile phone addiction (Su et al., 2018; Chen et al., 2021). Thus, mobile phone addiction may be induced by interpersonal adjustment difficulties resulting from preference for solitude. Third, the boredom emotion associated with solitude is also closely related to problematic mobile phone use. Individuals who are used to being alone tend to experience higher levels of boredom (Huang et al., 2010). Empirical evidence has also confirmed that boredom proneness can positively predict mobile phone addiction (Yang X. J. et al., 2020). Therefore, the experience of boredom induced by solitude may also lead to mobile phone addiction. In summary, considering that the preference for solitude may lead to a higher risk of mobile phone addiction, we proposed the following hypothesis:

Hypothesis 1: Preference for solitude would positively associated with mobile phone addiction among Chinese college students.

Considering the potential detrimental effects of preference for solitude on mobile phone addiction, it is necessary to further explore the mediating and moderating mechanisms underlying the association between preference for solitude and mobile phone addiction. Previous studies have found that psychological distress is an important link between individuals’ negative personality traits and behavioral problems (Yang X. J. et al., 2020). Therefore, introducing psychological distress to explore its mediating role in the relationship between preference for solitude and mobile phone addiction could help us understand the emotional mechanisms that preference for solitude induces mobile phone addiction. Furthermore, given that mindfulness is an important buffer for negative personality traits that induce behavioral and emotional maladjustment (Kim et al., 2015), the present study introduces mindfulness to explore its moderating role in the above mediating model. This could help us gain insight into the boundary conditions of preference for solitude inducing psychological distress and mobile phone addiction. Therefore, the present study would investigate psychological distress as a possible mediator and mindfulness as a possible moderator in the relationship between preference for solitude and mobile phone addiction.

### The Mediating Role of Psychological Distress

Psychological distress refers to general emotional disturbance related to depression, stress, anxiety as well as other emotional adaptation problems, which reflects the individual’s mental health status (Wong et al., 2014; Bore et al., 2016; Liu et al., 2019). A growing body of research has revealed that college students experience higher levels of psychological distress compared to the general population, leading to a range of negative consequences (Bore et al., 2016). According to the Compensatory Internet Use Theory (CIUT; Kardefelt-Winther, 2014), when people encounter psychosocial problems (e.g., depression, anxiety, and stress) in real life, they may turn to online world to alleviate the negative feelings. For example, a systematic review of longitudinal research indicated that psychological distress, such as anxiety and depression, was found to be predictive of problematic internet use (Anderson et al., 2016). As the most convenient, accessible and multi-functional carrier of mobile Internet technology, mobile phone has become a desirable tool for people to relieve stress and vent negative emotions (Yang X. et al., 2020; Yang X. F. et al., 2020), which might lead to mobile phone addiction in the long run (Liu et al., 2019). Consistent with this perspective, numerous empirical research has found that psychological distress is not only positively correlated with mobile phone addiction (Cho et al., 2017; Liu et al., 2019; Yang X. F. et al., 2020) but also a risk factor for the development of mobile phone addiction (Kim et al., 2015; Matar Boumosleh and Jaalouk, 2017). For instance, Liu et al. (2019) showed that psychological distress could positively predict mobile phone addiction. A meta-analysis research further indicated that individuals with depression or anxiety are more likely to use mobile phones and have a high risk of mobile phone addiction (Zhang et al., 2019). Besides, the model of deficient self-regulation characterization of problematic internet use (Tokunaga and Rains, 2010) also demonstrated that psychosocial problems could contribute in varying degrees to the development of mobile phone addiction. Specifically, individuals with psychological distress are unable to control their mobile phones use, which results in greater time spent on the mobile phones and increases the risk of mobile phone addiction (Tokunaga and Rains, 2010). Empirical studies further indicated that as stress, depression and anxiety increased, people’s ability of self-control faltered (Liu et al., 2016), leading to increased levels of mobile phone addiction (Cho et al., 2017).

In addition, higher levels of preference for solitude may be associated with greater psychological distress, especially in collectivistic culture (Chen and Zhou, 2012; Liu et al., 2015). There is growing evidence that preference for solitude is a significant vulnerability factor for individuals’ psychological maladjustment in China (Liu et al., 2014, 2015; Wang, 2016). For instance, Wang et al. (2013b) showed that preference for solitude was associated with greater anxiety, depression as well as emotion dysregulation in early adolescence. A longitudinal study conducted by Chen and Zhou (2012) further demonstrated that preference for solitude could positively predict depressive symptoms over time. Preference for solitude may lead to psychological distress for many reasons. According to the contextual-developmental perspective (Chen et al., 2006), social interaction, including mutual evaluations and responses, plays an important role in the influence of culture on human beings’ social and psychological adaption (Liu et al., 2015). In the context of collectivistic societies, people who intentionally do not engage in social interaction may evoke particularly negative evaluations and feedback from peers and society (Ding et al., 2015). Individuals with high preference for solitude may internalize both society’s and their peers’ negative views of unsociability and develop negative self-perceptions (Liu et al., 2014) and lower sense of self-worth (Bullock et al., 2020), which in turn causes psychological distress (ØStefjells et al., 2017). Thus, psychological distress may be induced by negative social evaluation resulting from preference for solitude. Besides, in terms of interpersonal risk, individuals with high preference for solitude are more likely to be engaged in interpersonal difficulties (Liu et al., 2014; Ding et al., 2018). For example, preference for solitude has been proved to trigger peer exclusion (Ooi et al., 2018), peer victimization (Wang et al., 2013a) as well as less peer preference (Zhang and Eggum-Wilkens, 2018). These negative life events were positively associated with stress, depression, and anxiety (Ladd et al., 2018). An empirical study also found that preference for solitude could predict psychological maladjustment through peer difficulties among Chinese children (Bullock et al., 2020). Therefore, individuals with high preference for solitude may experience more psychological distress owing to interpersonal difficulties.

Furthermore, psychological distress (e.g., depression) has been validated to play a mediating role in the relation between boredom proneness and problematic mobile phone use (Yang X. J. et al., 2020). A prior study also demonstrated that psychological distress could function as a mediator in the association between personality traits (e.g., trait procrastination) and mobile phone addiction (Yang X. F. et al., 2020). As far as this study is concerned, individuals with a high preference for solitude may experience more anxiety, depression, and stress, which, in turn, may lead them to have a higher risk of mobile phone addiction. Taking these considerations together, we proposed the following hypothesis:

Hypothesis 2: Psychological distress may act as a mediator in the link between preference for solitude and mobile phone addiction.

### The Moderating Role of Mindfulness

As mentioned above, preference for solitude may lead to increased mobile phone addiction via psychological distress. However, individuals with the same level of preference for solitude do not necessarily have the same level of psychological distress and the same risk of mobile phone addiction. Other individual characteristics may also contribute to the heterogeneity of outcomes. Drawing on the risk-buffering hypothesis, some protective personal factors can weaken the link between risk factors and psychosocial maladjustment and contribute to the development of individuals (Luthar et al., 2015). In this study, we would further introduce mindfulness as a beneficial individual trait and investigate whether the relationship between preference for solitude and mobile phone addiction can be buffered by mindfulness.

Mindfulness refers to the awareness state of being open, attentive, non-judgmental and acceptive to what is taking place in the present moment (Brown and Ryan, 2003; Kabat-Zinn, 2003). Considering that the level and propensity of mindfulness people hold varies from person to person, mindfulness can also be defined as a personality trait (Brown and Ryan, 2003; Wang G. et al., 2017). As a positive individual trait, numerous empirical studies have confirmed that mindfulness benefits individuals’ psychosocial adjustment, including self-esteem (Michalak et al., 2011), psychological well-being (Bajaj et al., 2016), and emotional regulation (Tomlinson et al., 2018). Research has also shown that high levels of mindfulness not only contribute to alleviating psychological distress (Khoury et al., 2015) but also could protect against the development of addictive behaviors such as mobile phone addiction (Elhai et al., 2018; Kim et al., 2018). Furthermore, mindfulness has been documented as an important protective factor that can buffer the adverse impacts of risk factors on individuals’ psychological and behavioral adaptation (Bergin and Pakenham, 2016; Yang et al., 2019; Lian et al., 2021b). For instance, Davis et al. (2016) showed that mindfulness could moderate the undesirable effect of insecure attachment on psychological distress including stress, depression, and anxiety, with this effect becoming weaker for individuals with high mindfulness. Liu et al. (2018) also demonstrated that the relationship between perceived stress and mobile phone addiction was alleviated for individuals with high mindfulness. Therefore, it could be inferred that mindfulness may also act as a moderator on the direct relationship between preference for solitude and mobile phone addiction and the indirect relationship through psychological distress.

Firstly, mindfulness may buffer the adverse effect of preference for solitude on mobile phone addiction. According to the re-perceiving model of mindfulness (Shapiro et al., 2006), through mindfulness practice, individuals are able to re-perceive their own internal and external experiences in every moment objectively and clearly, and accept their experience with great tolerance, which contributes to psychosocial adaption. Besides, high levels of mindfulness can also help individuals to get rid of automatic emotion and unhealthy behavior patterns (Brown and Ryan, 2003) and facilitate adaptive and flexible responses to negative stimulation in individuals (Shapiro et al., 2006). Therefore, mindfulness may have a risk-buffer effect on the relations between risk factors (e.g., preference for solitude) and psychosocial maladjustment outcomes (e.g., mobile phone addiction). That is, people with high levels of mindfulness may benefit more from an open and intentional state of consciousness when being alone. In contrast, those with low levels of mindfulness may involve in other activities to distract their attention when being alone, such as using mobile phones, and may lead to mobile phone addiction. In addition, previous studies have found that individuals with high preference for solitude often experience more loneliness (Liu et al., 2015), which has been proved to be a risk factor of mobile phone addiction (Shen and Wang, 2019). A systematic review and meta-analysis study conducted by Teoh et al. (2021) also showed that mindfulness intervention can alleviate the levels of loneliness. Thus, when experiencing loneliness resulting from preference for solitude, individuals with a high levels of mindfulness will be more able to accept and enjoy the state of loneliness at the moment, resulting in a lower risk of mobile phone addiction than those with a low levels of mindfulness. That is, the relationship between preference for solitude and mobile phone addiction becomes weaker with increases in mindfulness. Therefore, in accordance with the characteristics of mindfulness and the results of empirical studies, mindfulness might act as a buffer in the link between preference for solitude and mobile phone addiction.

Secondly, mindfulness may also alleviate the influence of preference for solitude on psychological distress. A substantial body of empirical studies have confirmed that mindfulness could promote individuals’ psychological adjustment. For instance, people with higher levels of mindfulness not only have a greater ability to regulate negative emotional states (Harnett et al., 2016) but also have lower levels of psychological distress (Drake et al., 2017). Besides, mindfulness has also been identified as an alleviator in the links between negative factors and psychological distress (Bajaj et al., 2016; Liu et al., 2017b; Lian et al., 2021b). For example, Lian et al. (2021b) indicated that individuals with high levels of mindfulness experience less psychological distress in contrast to those with low mindfulness when they are alone. Thus, mindfulness may buffer the negative effect of preference for solitude on psychological distress. Furthermore, mindfulness can also facilitate choices that are congruent with one’s actual needs (Brown and Ryan, 2003; Wang G. et al., 2017), which may be another reason why mindfulness could alleviate psychological distress in individuals with high preference for solitude. Based on the values clarification mechanism of the re-perceiving model of mindfulness, individuals can re-perceive the values that are constrained by family, culture, and society through mindfulness practice, which in turn helps them to choose the values consistent with personal needs (Shapiro et al., 2006). Therefore, although preference for solitude is contrary to collectivistic values in group-oriented societies, high levels of mindfulness may help individuals to re-perceive and accept their choices, and experience less psychological distress. Prior studies further documented that mindfulness could act as a moderator in the relations between other personality traits and psychological distress (Hamill et al., 2015; Harnett et al., 2016). For instance, a study conducted by Drake et al. (2017) found that the association between neuroticism and non-specific psychological distress was alleviated for individuals with high mindfulness. Therefore, mindfulness may act as an alleviator in the relationship between negative personality traits and mobile phone addiction. Above all, we propose the hypothesis as follows:

Hypothesis 3: Mindfulness would moderate the relationship between preference for solitude and psychological distress as well as mobile phone addiction, with the relationship being weaker for individuals with high levels of mindfulness.

### The Present Study

**FIGURE 1:**
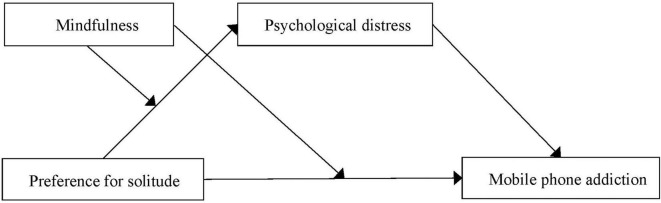
The proposed moderated mediation model.

Considering the adverse impacts of preference for solitude in the context of collectivistic societies and its possible negative effect on mobile phone addiction, it is imperative to examine the relation between preference for solitude and mobile phone addiction and further explore its potential mechanisms. In the present study, we focus on the mediating role of psychological distress and the moderating role of mindfulness in the relationship between preference for solitude and mobile phone addiction. Specifically, this study aimed to examine: (a) whether preference for solitude would positively associate with mobile phone addiction; (b) whether psychological distress would mediate the relationship between preference for solitude and mobile phone addiction; (c) whether mindfulness would moderate the link between preference for solitude and mobile phone addiction, and (d) whether mindfulness would moderate the mediating effect of psychological distress. Taken together, we proposed a moderated mediation model which can address questions regarding mediation (i.e., how preference for solitude relates to mobile phone addiction) and moderation (i.e., when and for whom the connection becomes stronger or weaker) simultaneously. The proposed model was illustrated in Figure 1.

## Materials and Methods

### Participants

In this study, data were collected through convenience sampling from a comprehensive university in Jingzhou, a major city located in central China. Our survey was conducted on a class basis, and a total of 958 students in 20 classes of three grades agreed to participate in our study. After removing participants who had poor task performance in the study (e.g., provided incomplete information, extreme outliers, and failed an attention check), there were 927 participants in the final sample (effective recovery 96.74%). Among them were 371 (40.03%) males and 556 (59.97%) females. Their mean age was 19.89 years old (*SD* = 1.22), with ages ranging from 16 to 24 years old. Two hundred and ninety-eight (32.10%) of them were freshmen (grade 1); and three hundred and twenty-nine (35.50%) of them were sophomores (grade 2); three hundred (32.40%) of them were juniors (grade 3). Furthermore, the average number of years that participants use mobile phones was 5.91 years (*SD* = 2.51, range = 1–15 years), and the average number of hours per day that participants use their mobile phones was 6.39 h (*SD* = 2.75, range = 1–16 h). In terms of how often the participants check their phones everyday, two hundred and sixty (28.05%) of them checked their phones every 10 min; four hundred and eight (44.01%) of them checked their phones every half hour; and two hundred and fifty-nine (27.94%) of them checked their phones every hour or more.

### Procedure

The study was approved by the Ethics Committee for Scientific Research at the authors’ institution prior to data collection. Researchers randomly selected six to seven classes from the first to the third grades in the target university to participate in this survey. We first contacted all the class advisers and asked for their permission. Furthermore, we recruited graduate students majoring in psychology for training, and they cooperated with the researchers to distribute and collect the questionnaires. The data were collected in the classrooms, and the researchers first explained the purpose of the study to all students. Students who had experiences of using mobile phones and volunteered to participate in the survey raised their hands and signed the written consent form for this study. Participants were informed that they could terminate their participation at any time during the survey without any repercussion. Meanwhile, researchers emphasized that the data collected were used for academic research only, and stressed the principles of anonymity, independence, and confidentiality of this survey. During the survey, the classroom remained quiet. All participants completed the manuscript-and-pencil survey within 30 min. After the survey was completed, researchers expressed their gratitude to all participants and teachers and informed that the results of the study would be published in the form of a scientific article on the website of the Department of Psychology.

### Measurements

#### Preference for Solitude

Preference for solitude was measured by the Chinese version of the preference for solitude scale (Chen and Zhou, 2012), which was revised from the original version developed by Burger (1995). The Chinese version of preference for solitude scale has been widely used in Chinese college students with good reliability and validity (Zhang et al., 2018). The scale consists of 11 items, each of which adopts the forced-choice method (e.g., “I enjoy being by myself vs. I enjoy being around people”). Participants were asked to choose between two mutually exclusive options, one reflecting a preference for solitude (record as 0 point) and the other reflecting a preference for being with other people (record as 1 point). Responses were averaged to form a measure of students’ preference for solitude, with higher scores indicating higher levels of preference for solitude tendency. In the current study, the items demonstrated acceptable reliability (Cronbach’s α = 0.75).

#### Psychological Distress

We used the Chinese version of Depression Anxiety Stress Scale-21 (DASS-21; Wang et al., 2016) to assess participants’ psychological distress, which has showed good reliability and validity in Chinese sample (Lian et al., 2021a). The scale consists of 21 items that cover three subscales: depressive symptoms scale (e.g., “I couldn’t seem to experience any positive feeling at all”), anxiety symptoms scale (e.g., “I was aware of dryness of my mouth”), and stress symptoms scale (e.g., “I found it hard to wind down”). All items were rated on a four-point scale ranging from 0 (did not apply to me at all) to 3 (applied to me very much or most of the time). Higher scores reflect more serious psychological distress. Cronbach’s alpha coefficient for the DASS-21 was 0.94.

#### Mindfulness

The study used the Chinese version of the 10-item Child and Adolescent Mindfulness Measure (CAMM) to measure the level of the participants’ mindfulness, which was originally developed by Greco et al. (2011), and later revised by Liu et al. (2018). Participants responded to the 10 items on a five points Likert-type scale ranging from 1 (*never*) to 4 (*always*) (e.g., “I walk from class to class without noticing what I’m doing”). All of the responses were averaged after reversing the reverse-scored items to form a measure, such that higher scores represented higher levels of mindfulness in everyday life. Previous research has shown that the 10-item Child and Adolescent Mindfulness Measure was of satisfactory reliability and validity in both Chinese adolescents (Yang et al., 2019; Lian et al., 2021a) and college students (Yang et al., 2021). In order to further verify the validity of the scale in this study, confirmatory factor analysis was conducted. The results showed that the scale had good structural validity: χ^2^/*df* = 3.681, RMSEA = 0.05, CFI = 0.96, NFI = 0.94, GFI = 0.98. The items also demonstrated good reliability in the current study (Cronbach’s α = 0.77).

#### Mobile Phone Addiction

As has been widely used in a diversity of samples to measure individuals’ mobile phone addiction tendency, the Mobile Phone Addiction Index (MPAI; Leung, 2008) was used in this study. The scale has been proved to have good reliability and validity in Chinese students and young adults (Chen et al., 2016; Liu et al., 2017b). This scale consists of seventeen items (e.g., “You have attempted to spend less time on your mobile phone but are unable to”) that cover four dimensions including anxiety and feeling lost, inability to control cravings, withdrawal and escape, as well as productivity loss. Participants were asked to respond to all items on a five-point Likert-type scale ranging from 1 for “never” to 5 for “always,” with higher scores indicating more severe mobile phone addiction. In the present study, Cronbach’s α was 0.84.

#### Control Variables

Gender, age, grade, years of using mobile phones, as well as time and frequency spending on mobile phones per day were included as control variables in our model, as previous studies found that they were closely associated with the main variables in this study (Lian et al., 2021a; Yang et al., 2021).

#### Data Analysis

In this study, a moderated mediation model was constructed with preference for solitude as the independent variable, mobile phone addiction as the dependent variable, psychological distress as the mediating variable, and mindfulness as the moderating variable. Statistical analyses were conducted using SPSS 25.0. Since self-report data were collected for the present study, Harman single factor test was conducted to test the potential common method biases before data processing (Podsakoff et al., 2003). A total of 59 items of four main variables were tested. The results showed that twelve factors had eigenvalues greater than 1. These factors contributed 55.31% of the total variance. The first factor explained only 19.91% of the total variance, which did not reach the critical criterion of 40% (Zhou and Long, 2004), indicating that there was no significant common method bias in the present study.

After common method bias evaluation, we carried out the following data processing steps. Firstly, we employed descriptive statistics and Pearson correlation analysis to examine the means, standard deviations, and bivariate associations of the study variables. Secondly, an independent sample *t*-test was adopted to examine the gender differences for main variables; one-way ANOVA was applied to examine differences in grade level and differences in frequency of mobile phones use for the main variables. **Thirdly**, sobel test^1^ was employed to examine the predicted indirect effect of psychological distress in the relationship between preference for solitude and mobile phone addiction. The absolute value of z- value greater than 1.96 indicates that the mediating effect is significant. **Fourthly**, the SPSS macro PROCESS (Model 8) Version 3.3^2^ suggested by Hayes (2013) was used to test the moderated mediation model. This SPSS macro has been used to test mediating and moderating models in several studies, in which this SPSS macro showed higher statistical testability (Yang X. J. et al., 2020; Lian et al., 2021a,b). The proposed moderated mediation model, name as Model 8, tests the influence of a moderator on a mediation model, with the moderation occurring on the direct path (the relationship between the independent variable and dependent variable) and the first half of the indirect path (the relationship between the independent variable and the moderator) of the mediation model. Specifically, we used Model 8 to test the moderating effect of mindfulness on the direct relationship between preference for solitude and mobile phone addiction, and the indirect relationship through psychological distress (the link between preference for solitude and psychological distress). Bootstrap confidence intervals (95% CIs) were applied to determine whether the regression coefficients in Model 8 were significant from 5,000 random samples of the data. CIs excluding zero indicated significant effects. Furthermore, simple slopes analyses were performed to decompose all the potential significant interaction effects (Toothaker, 1994).

## Results

### Preliminary Analyses

Table 1 presented the means, standard deviations, and correlations for all of the observed variables. As hypothesized, preference for solitude was positively correlated with both psychological distress (*r* = 0.23, *p* < 0.01)and mobile phone addiction (*r* = 0.22, *p* < 0.01), and negatively correlated with mindfulness (*r* = −0.22, *p* < 0.01). Psychological distress was positively correlated with mobile phone addiction (*r* = 0.32, *p* < 0.01) and negatively correlated with mindfulness (*r* = −0.34, *p* < 0.01). Mindfulness was negatively correlated with mobile phone addiction (*r* = −0.46, *p* < 0.01). Furthermore, gender was positively correlated with both preference for solitude (*r* = 0.10, *p* < 0.01) and mobile phone addiction (*r* = 0.15, *p* < 0.01), and negatively correlated with both psychological distress (*r* = −0.11, *p* < 0.01) and mindfulness (*r* = −0.07, *p* < 0.05). Years of using mobile phones was positively correlated with mobile phone addiction (*r* = 0.12, *p* < 0.01). Time spending on mobile phones per day was positively correlated with both preference for solitude (*r* = 0.12, *p* < 0.01) and mobile phone addiction (*r* = 0.17, *p* < 0.01), and negatively correlated with mindfulness (*r* = −0.12, *p* < 0.01). Frequency of using mobile phones per day was positively correlated with mindfulness (*r* = 0.09, *p* < 0.01) and negatively correlated with mobile phone addiction (*r* = −0.19, *p* < 0.01). Whereas, age and grade showed no significant correlation with all of the core observed variables.

**TABLE 1:** Descriptive statistics and interrelations among all of the observed variables.

Variables	*M*	*SD*	1	2	3	4	5	6	7	8	9	10
1. Gender	–	–	1									
2. Grade	–	–	0.15**	1								
3. Mobile phone usage frequency	–	–	−0.11**	–0.002	1							
4. Years of using mobile phone	5.91	2.51	−0.002**	0.14**	−0.10**	1						
5. Time spending on mobile phone per day	6.39	2.75	0.15**	0.08*	−0.35**	0.15**	1					
6. Age	19.89	1.22	–0.05	0.74**	0.03	0.14**	0.04	1				
7. Preference for solitude	0.51	0.26	0.10**	–0.02	–0.06	0.03	0.12**	–0.05	1			
8. Psychological distress	0.88	0.55	−0.11**	–0.04	0.01	0.01	0.06	–0.05	0.23**	1		
9. Mobile phone addiction	2.62	0.57	0.15**	0.004	−0.19**	0.12**	0.17**	–0.03	0.22**	0.32**	1	
10. Mindfulness	2.21	0.54	−0.07*	–0.01	0.09**	–0.05	−0.12**	0.04	−0.22**	−0.34**	−0.46**	1

N = 927. **p< 0.01, *p < 0.05.

Besides, Table 2 presented the differences of the observed variables in gender, Grade-level and frequency of mobile phones use. Results of independent-sample *t*-test indicated that there were significant gender differences in preference for solitude, psychological distress, mobile phone addiction, and mindfulness. Furthermore, the results of one-way ANOVA indicated that preference for solitude, mobile phone addiction, and mindfulness all showed no significant Grade-Level differences. Whereas only psychological distress showed a significant Grade-Level difference [*M*_grade2_ (1.00) > *M*_grade1_ (0.85); *M*_grade2_ (1.00) > *M*_grade3_ (0.79)]. In addition, the results of one-way ANOVA also indicated that preference for solitude and psychological distress showed no significant frequency differences. Whereas mobile phone addiction [*M*_10min_ (2.78) > *M*_30min_ (2.59); *M*_10min_ (2.78) > *M*_1h_ (2.49)] and mindfulness [*M*_1h_ (2.28) > *M*_10min_ (2.16)] showed a significant frequency differences.

**TABLE 2:** The differences of the observed variables in gender, Grade-level and frequency of mobile phones use.

Variables	*M* ± *SD*	Preference for solitude	Psychological distress	Mobile phone addiction	Mindfulness
Gender	Male	0.48 ± 0.31	0.95 ± 0.58	2.51 ± 0.59	2.26 ± 0.59
	Female	0.5 ± 0.23	0.84 ± 0.52	2.69 ± 0.56	2.18 ± 0.50
	*t*	−2.92**	3.15*	−4.71***	2.12*
Grade	Grade1	0.52 ± 0.27	0.85 ± 0.55	2.61 ± 0.53	2.21 ± 0.57
	Grade2	0.51 ± 0.29	1.00 ± 0.57	2.63 ± 0.60	2.23 ± 0.55
	Grade3	0.50 ± 0.23	0.79 ± 0.50	2.61 ± 0.59	2.19 ± 0.50
	*F*	0.24	12.75***	0.12	0.52
Frequency	10 min	0.52 ± 0.31	0.89 ± 0.56	2.78 ± 0.58	2.16 ± 0.53
	30 min	0.54 ± 0.24	0.87 ± 0.54	2.59 ± 0.54	2.20 ± 0.54
	1 h	0.48 ± 0.24	0.90 ± 0.54	2.49 ± 0.59	2.28 ± 0.56
	*F*	1.64	0.47	18.37***	3.50*

N = 927. *p< 0.05, **p < 0.01, and ***p < 0.001.

### Testing for the Proposed Moderated Mediation Model

**FIGURE 2:**
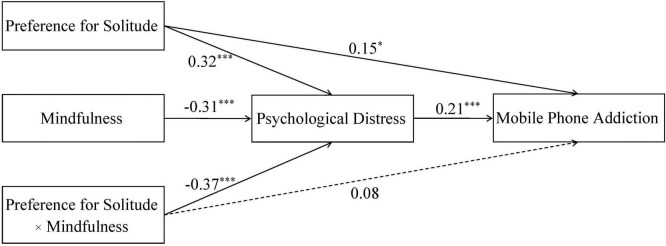
Moderated mediation analysis (*N* = 927). *p < 0.05, **p < 0.01, and ***p < 0.001.

Hayes’s (2013) macro PROCESS (Model 8) was adopted to examine the proposed moderated mediation model. Figure 2 and Table 3 presented the main results of the moderated mediation analysis. As expected, preference for solitude positively predicted psychological distress (*B* = 0.32, *p* < 0.001) and mobile phone addiction (*B* = 0.15, *p* < 0.05). Psychological distress positively predicted mobile phone addiction (*B* = 0.21, *p* < 0.001). Furthermore, sobel test was employed to examine the significance of the indirect effect of preference for solitude on mobile phone addiction via psychological distress. The results indicated that psychological distress significantly mediated the relationship between preference for solitude and mobile phone addiction (*z* = 5.93, *p* < 0.001). These results provided compelling evidence that preference for solitude was associated with an increase in mobile phone addiction and this relation was mediated by psychological distress. Thus, Hypothesis 1 and 2 were supported.

**TABLE 3:** Regression results for the conditional indirect effects (moderated mediation).

Model											
**Model 1: Total effect model (outcome variable: mobile phone addiction)**											
***R***	***R* ^2^**		***F***		***df*_1_**	***df*_2_**	***p***	***B***	***SE***	***t***	***p***
0.33	0.11		15.51		7	919	<0.001				
Constant								2.38***	0.42	5.66	<0.001
Gender								0.13***	0.04	3.33	<0.001
Age								–0.01	0.02	–0.40	>0.05
Grade								–0.01	0.03	–0.25	>0.05
Years of mobile phone use								0.02**	0.01	2.82	<0.01
Time spending on mobile phone per day								0.02**	0.01	2.19	<0.01
Mobile phone usage frequency								−0.10***	0.03	–3.95	<0.001
Preference for solitude								0.40***	0.07	5.85	<0.001

**Model 2: Mediator variable model (outcome variable: psychological distress)**											
***R***		***R* ^2^**		***F***	***df*_1_**	***df*_2_**	***p***	***B***	***SE***	***t***	***p***

0.42		0.17		21.49	9	917	< 0.001				
Constant								1.39***	0.39	3.61	<0.001
Gender								−0.15***	0.04	–4.28	<0.001
Age								–0.02	0.02	–0.93	>0.05
Grade								0.004	0.03	0.12	>0.05
Years of mobile phone use								–0.001	0.01	–0.10	>0.05
Time spending on mobile phone per day								0.01	0.01	0.86	>0.05
Mobile phone usage frequency								0.03	0.02	1.42	>0.05
Preference for solitude								0.32***	0.07	4.79	<0.001
Mindfulness								−0.31***	0.03	–9.81	<0.001
Preference for solitude × Mindfulness								−0.37***	0.11	–3.39	<0.001

**Model 3: Dependent variable model (outcome variable: mobile phone addiction)**											
***R***		***R* ^2^**		***F***	***df*_1_**	***df*_2_**	***p***	***B***	***SE***	***t***	***p***

0.55		0.30		39.01	10	916	< 0.001				
Constant								2.08***	0.38	5.55	<0.001
Gender								0.15***	0.03	4.28	<0.001
Age								0.01	0.02	0.53	>0.05
Grade								–0.03	0.03	–0.93	>0.05
Years of mobile phone use								0.02**	0.01	2.75	<0.01
Time spending on mobile phone per day								0.01	0.01	1.53	>0.05
Mobile phone usage frequency								−0.10***	0.02	–4.20	<0.001
Preference for solitude								0.15*	0.06	2.37	>0.05
Psychological distress								0.21***	0.03	6.44	<0.001
Mindfulness								−0.38***	0.04	–11.82	<0.001
Preference for solitude × Mindfulness								0.08	0.11	0.74	>0.05

**Conditional indirect effect analysis at values of mindfulness (M ± SD)**											
								***B***	**Boot SE**	**Boot LLCI**	**Boot ULCI**

M − 1SD (1.67)								0.11	0.03	0.06	0.17
M (2.21)								0.07	0.02	0.03	0.11
M + 1SD (2.75)								0.02	0.02	–0.01	0.07

N = 927. Bootstrap sample size = 5000. R^2^, coefficient of determination; df, degree of freedom; B, unstandardized regression coefficients; SE, standard error; CIs, bootstrap confidence intervals; LLCI, lower limit of the 95% confidence interval; ULCI, upper limit of the 95% confidence interval. *p < 0.05, **p < 0.01, and ***p < 0.001.

In order to examine Hypothesis 3, two interaction effects were also analyzed with macro PROCESS (Model 8) by Hayes (2013). There was a significant preference for solitude × mindfulness interaction effect on psychological distress (*B* = −0.37, *p* < 0.001) in mediator variable model. Preference for solitude × mindfulness interaction was not significant on mobile phone addiction (*B* = 0.08, *p* > 0.05) in the dependent variable model. These findings indicated that only the association between preference for solitude and psychological distress was moderated by mindfulness.

**FIGURE 3:**
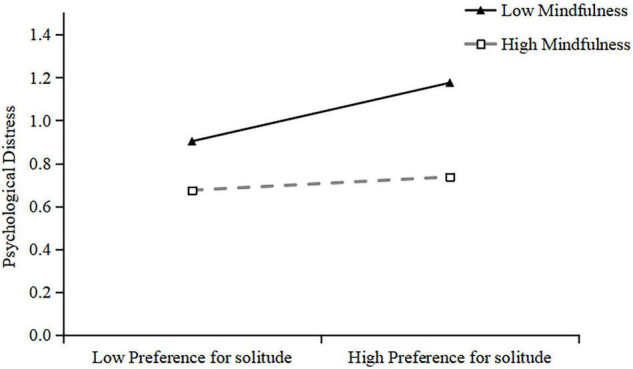
Mindfulness moderated the relationship between preference for solitude and psychological distress.

Additionally, simple slope analyses were conducted to illustrate this significant interaction and explore whether slopes for the high-mindfulness group (1 *SD* above the mean) were different from slopes for the low-mindfulness group (1 *SD* below the mean) in the mediator variable model. The results were plotted in Figure 3. As shown in Figure 3, the effect of preference for solitude on psychological distress was positive and significant for college students with low mindfulness (*B* = 0.52, *t* = 6.66, *p* < 0.001), whereas it was not significant for those with high mindfulness (*B* = 0.12, *t* = 1.20, *p* > 0.05). The results indicated that the indirect effect of psychological distress in the relationship between preference for solitude and mobile phone addiction was stronger for college students with lower mindfulness. However, no matter what levels of mindfulness are, preference for solitude could significantly predict mobile phone addiction among college students.

## Discussion

In the era of information technology, mobile phones have been regarded as a necessity in the lives of college students (Lepp et al., 2014). With the increasing incidence of mobile phone addiction among college students, the potential risk factors for mobile phone addiction has attracted more and more researchers’ attention. Although various personality trait factors have been proven to be significant predictors of mobile phone addiction (Yang X. et al., 2020), limited attention has been paid to preference for solitude. In addition, the majority of the research on after-effects of preference for solitude has been limited to individualistic contexts, with fewer studies in China and other non-Western cultures (Wang, 2016). To fill these gaps, this study was designed to examine the relation between preference for solitude and mobile phone addiction and its underlying mechanisms among Chinese college students. Specifically, we proposed a moderated mediation model to analyze the role of psychological distress and mindfulness in the relation between preference for solitude and mobile phone addiction. Correlation analyses revealed that preference for solitude was positively related to mobile phone addiction among Chinese college students. Hypothesis 1 was supported. Furthermore, the mediation analyses uncovered a mediating role of psychological distress in the association between preference for solitude and mobile phone addiction. Hypothesis 2 was supported. In addition, the moderated mediation analyses showed that the indirect effect of preference for solitude on mobile phone addiction was moderated by mindfulness. However, mindfulness could not moderate the direct relation between preference for solitude and mobile phone addiction. In other words, college students with higher levels of mindfulness could successfully alleviate the adverse impacts of preference for solitude on psychological distress, which further reduce mobile phone addiction. Hypothesis 3 was partially supported. Findings enlightened us that we could attenuate the potential adverse effects resulting from preference for solitude on psychological distress by enhancing the levels of mindfulness, so as to reduce the risk of mobile phone addiction.

First, in line with previous research documenting that preference for solitude has a negative impact on individuals’ psychosocial adaptation in collectivistic culture (Liu et al., 2015), the present study indicated that preference for solitude could positively and significantly predict mobile phone addiction among Chinese college students. This finding further illustrated that there are not only individual differences in the preference for solitude but also cultural differences in its after-effects. Specifically, the psychological and social adaptive functions of preference for solitude may depend on the cultural and social context in which the individual lives. Compared with individualistic societies, collectivistic societies emphasize social affiliation and encourage individuals to interact with society and others (Liu et al., 2014; Ding et al., 2015). Chinese college students with high preference for solitude may thus experience psychosocial maladjustment resulting from their preferences and behaviors that are contrary to social values, which was a significant predictor of mobile phone addiction (Cho et al., 2017; Yang X. F. et al., 2020). Furthermore, our findings also coincide with the uses and gratification theory (Parker and Plank, 2000) proposing that satisfaction of psychological needs is an important trigger for mobile phone addiction. Individuals with high preference for solitude often suffer from a series of interpersonal problems and even social isolation in collectivistic societies (Liu et al., 2014, 2015). These negative experiences of interpersonal interactions will lead to unsatisfied basic psychological needs of individuals, such as the need to belong and escapism (Xu et al., 2021). In this case, individuals will turn to online world where such needs can be met (Parker and Plank, 2000). Based on the multiple functions of mobile phones, college students often tend to rely on them to escape from unpleasant interpersonal experiences in real lives, and to obtain the sense of satisfaction and pleasure through using them, which eventually leads to mobile phone addiction. Previous studies also demonstrated that preference for solitude has been proved to positively predict interpersonal difficulties in collectivistic societies (Liu et al., 2014; Ding et al., 2018), which can further positively predict mobile phone addiction (Su et al., 2018; Chen et al., 2021). Therefore, Chinese college students with high preference for solitude may have higher risks for mobile phone addiction.

Second, consistent with prior research showing that the mediating role of psychological distress (e.g., stress and depression) in the relationship between undesirable personality traits and problematic mobile phone use (Yang X. F. et al., 2020; Yang X. J. et al., 2020), this study further indicated that psychological distress could significantly mediate the relationship between preference for solitude and mobile phone addiction. In other words, Chinese college students with high preference for solitude tend to experience psychological distress that subsequently leads to mobile phone addiction. Although a considerable amount of research has supported the correlation between psychological distress and mobile phone addiction (Liu et al., 2019; Yang X. F. et al., 2020) as well as preference for solitude and psychological distress (Chen and Zhou, 2012; Wang et al., 2013b), to our knowledge, the present study is the first to investigate the mediating role of psychological distress between preference for solitude and mobile phone addiction. At the same time, our findings further verified that preference for solitude exerted a negative effect on individuals’ psychological and behavioral adaption in collectivistic societies like China. Furthermore, this study also indicated that the internalizing difficulties (e.g., psychological distress) induced by preference for solitude can further lead to the externalizing difficulties (e.g., mobile phone addiction) of individuals.

According to the Ecological System Theory (Bronfenbrenner and Ceci, 1994), the development of individuals’ socialization and social emotional functions is constrained by cultural macro systems. Individualistic cultures emphasizes that the goal of individuals’ socialization is independence as well as autonomy (Chen and French, 2008; Ding et al., 2015). On the contrary, collectivistic culture emphasizes that individuals should form a sense of social belonging, care for others and make their own contributions to the collective (Chen and Zhou, 2012; Liu et al., 2014). Therefore, preference for solitude has a relatively mild adaptive function in the individualistic societies, whereas it is detrimental to the psychological adaptation (e.g., psychological distress) for individuals in collectivistic cultures. Previous studies of Chinese samples have also found that people with a higher preference for solitude experienced more psychological distress (Wang et al., 2013b; Ding et al., 2015). Furthermore, our findings were also in accordance with the contextual-developmental perspective (Chen et al., 2006) suggesting that social evaluations and responses guided by cultural norms and values could influence individuals’ psychosocial development. In collectivistic societies, people with a high preference for solitude may be seen as unsociable, and they are more likely to be evaluated negatively and therefore being excluded or isolated. These negative experiences could lead to a series of psychological distress, including depressive symptoms, anxiety, and stress (Wang et al., 2013b; Ladd et al., 2018; Bullock et al., 2020). In addition, according to the Psychological Decompensation Hypothesis (Gao and Chen, 2006) and Compensatory Internet Use Theory (Kardefelt-Winther, 2014), psychosocial problems, such as psychological distress, are one of the basic motivations for individuals’ use of Internet or mobile phones. That is, when individuals with high preference for solitude experience negative emotions, such as stress, depression and anxiety, they may cope with and alleviate their negative feelings through compensatory mobile phones use. Unfortunately, once they get relief from negative emotions through mobile phones use, they will have a strong desire to spend more time on mobile phones, which in the long run can lead to increased risks in mobile phone addiction. Therefore, psychological distress induced by preference for solitude may lead to mobile phone addiction among Chinese college students. Moreover, the test of the mediating effect of psychological distress further supported the Interaction of Person-Affect-Cognition-Execution (I-PACE) model (Brand et al., 2019). According to this theory, the development and maintenance of addictive behaviors (e.g., mobile phone addiction) is the consequence of the interactions between core personality trait (e.g., preference for solitude) and affective (e.g., depression, anxiety, and stress). Overall, psychological distress was an underlying mechanism for us to understand how preference for solitude influences mobile phone addiction.

Additionally, one more important finding in the present study was that mindfulness could moderate the relationship between preference for solitude and psychological distress. Specifically, the indirect effect that preference for solitude exerted on mobile phone addiction via psychological distress is moderated and buffered by mindfulness, with this effect being stronger for college students with lower levels of mindfulness. This result might indicate that mindfulness, as a positive personality trait, could help us alleviate the adverse effect of negative personality traits on mental health. With the psychological stress ameliorated, the risk of phone addiction will be reduced. Our findings were consistent with the model of Mindfulness De-automatization (Kang et al., 2013) and previous studies confirming that mindfulness is an important protective factor for individuals’ psychological health (Harnett et al., 2016; Tomlinson et al., 2018; Lian et al., 2021b). According to the model of Mindfulness De-automatization (Kang et al., 2013), the de-automatizing function of mindfulness can reduce individuals’ automatic cognitive and emotional reaction toward the internal and external stimuli, and facilitate flexible and adaptive responding to negative stimuli, which has implications for mental health. When college students with high mindfulness suffer from social exclusion and isolation in collectivistic societies induced by preference for solitude, they may accept their situation and feelings realistically and adjust their response style flexibly, so as to reduce psychological distress. This further reduces individuals’ automatic behavior of using mobile phones to relieve negative emotions, and reducing the risk of mobile phone addiction. Furthermore, our results also coincide with the values clarification mechanism of the re-perceiving model of mindfulness (Shapiro et al., 2006), which suggests that re-perceiving can help people to separate from the values defined by culture and society, and reflect upon them with greater clarity and objectivity. Mindfulness thus facilitates individuals to choose values that are consistent with their real needs and to accept these values that are inconsistent with mainstream values (Brown and Ryan, 2003; Shapiro et al., 2006). Therefore, Chinese college students with high levels of mindfulness generally hold positive attitudes toward their choices, and they can not only respect and accept their preference for solitude but also benefit more from it. Unfortunately, those with low levels of mindfulness might not be able to cope well with the fact that their preferences for solitude are inconsistent with mainstream, thus they may be trapped in psychosocial maladjustment, leading to psychological distress and in turn mobile phone addiction.

At last, another result that deserves our attention is that the direct effect of preference for solitude on mobile phone addiction was not moderated by mindfulness. In other words, preference for solitude can significantly and positively predict mobile phone addiction among college students, regardless of individuals’ levels of mindfulness. This may be related to the mobile phones usage habits of individuals with high preference for solitude. In the era of mobile Internet, a “natural connection” has gradually formed between people and mobile phones. As the main users, college students are inseparable from mobile phones (Long et al., 2016). On the one hand, with the rapid development of e-commerce in recent years, college students have been widely accepted and got used to using mobile phones for online transactions, such as online payment, shopping, and transfer (Jiang and Zhao, 2016), making mobile phone an indispensable product for every college student. On the other hand, with its mobile, multi-functional, and multi-modal nature, mobile phone is usually an ideal device for college students to spend their time anytime and anywhere (Yang X. et al., 2020). It’s worth noting that college student with high preference for solitude are used to spending time alone and avoiding social interaction. As a result, they usually have more leisure time (Leung, 2015) and will experience higher levels of boredom (Huang et al., 2010), which leads them to turn to mobile phones for leisure and entertainment habitually when they being alone. Over time, solitude forms a “natural connection” with the phone, which leads college students with high preference for solitude to have a high risk of mobile phone addiction. In this case, although mindfulness can help individuals become aware and re-perceive of the present moment, it doesn’t prevent individuals from using their mobile phones. Both individuals with high and low levels of mindfulness used their phones as a way to pass the time when they being alone. Therefore, mindfulness did not mitigate the effect of preference for solitude on mobile phone addiction. In summary, although the results were not in line with our expectations, it also suggests that preference for solitude preference is a significant and powerful predictor of mobile phone addiction among Chinese college students, which should not be ignored.

### Limitations and Implications

Although the present study provides valuable findings for us to understand how and when preference for solitude influences Chinese college students’ psychological distress and mobile phone addiction, several limitations in this study should be taken into account.

First, this study utilized convenience sampling to collect data only from a university in China, and the representativeness of the sample remains to be verified. Therefore, caution should be used when generalizing the findings of this study to other groups. Future research could use random or stratified sampling to examine the model in diverse and large groups, especially in other countries/regions in different cultures. Second, due to our cross-sectional study design, causality cannot be established. Future studies could adopt experimental or longitudinal design to further examine the causal relationship between preference for solitude, psychological distress, and mobile phone addiction. Third, this study only measured the levels of individuals’ mindfulness trait and did not conduct mindfulness training (e.g., mindfulness-based cognitive treatment) to explore the role of mindfulness. Considering the buffering and protective effects of mindfulness traits on psychological distress in the present study, it is also necessary to conduct the intervention research. Future studies could examine the role of mindfulness in the pathway of preference for solitude influencing psychological distress and mobile phone addiction by enhancing mindfulness through mindfulness training. Finally, the data of the present study were collected only through self-reported questionnaires, which cannot avoid the influence of potential social desirability bias and common method bias on the research results. Future studies should allow for a multidimensional approach to collect more objective data, including peer nominations, parent reports, and school records.

Despite the limitations above, the present study deepens previous research by revealing the relationship between preference for solitude and mobile phone addiction, as well as its underlying mediating and moderating mechanisms. First of all, this study, for the first time, confirmed the positive predictive effect of preference for solitude on mobile phone addiction in collectivistic societies like China. Secondly, this study is the first attempt to reveal the mediating role of psychological distress and the protective role of mindfulness to explain how and when preference for solitude leads to mobile phone addiction among Chinese college students.

In addition to the theoretical and empirical contributions discussed above, our study also has several important practical implications. Firstly, the results indicated that mindfulness was an important protective factor for individuals’ psychological distress. Higher levels of mindfulness mean that preference for solitude would have a weaker or even disappearing effect on psychological distress, leading to a decreased risk of mobile phone addiction. Therefore, how to guide college students with high preference for solitude to improve their levels of mindfulness deserves special attention. On the one hand, for college students with high preference for solitude, self-training of mindfulness can be carried out under the guidance of psychological experts through mindfulness training courses. For instance, John Lothes et al. (2019) adopted Dialectical Behavior Therapy’s (DBT’s) mindfulness skills and Mindfulness-based Stress Reduction practices over an 8-week period on college students. Results found that these specific mindfulness training could significantly increase the level of mindfulness and reduce negative emotions (e.g., general anxiety) among college students (John Lothes et al., 2019). On the other hand, for mental health education departments in universities, mindfulness-based group counseling can be conducted for individuals with high preference for solitude. Previous studies have found that mindfulness-based group counseling can improve college students’ ability to observe and perceive emotional reactions and significantly reduce negative emotions (Hu et al., 2017). In conclusion, the negative effects of preference for solitude can be buffered by activating or cultivating individuals’ mindfulness.

Secondly, considering that psychological distress plays a “bridge” role in the relationship between preference for solitude and mobile phone addiction, parents, and educators could help college students with high preference for solitude reduce the risk of mobile phone addiction by decreasing their psychological distress. In addition to improving awareness of one’s emotional state through mindfulness, individuals can also improve negative emotions through physical activity. Previous research has shown that physical activity has a positive impact on an individuals’ mental health (Downs and Strachan, 2016). College students who regularly participate in physical exercise have higher self-efficacy for emotion regulation and better emotion management strategies to effectively manage their emotions and avoid psychological distress (Jiang et al., 2018). Therefore, for college students with high preference for solitude, forming a good physical activity habit is also an effective strategy to improve psychological distress and reduce mobile phone addiction.

## Data Availability Statement

The raw data supporting the conclusions of this article will be made available by the authors, without undue reservation.

## Ethics Statement

The studies involving human participants were reviewed and approved by the Ethics Committee for Scientific Research of Yangtze University. Written informed consent to participate in this study was provided by the participants.

## Author Contributions

W-YC and LY were responsible for the overall development of this study, including the planning of sample collection, data analysis, writing, and polishing of the manuscript. Y-RY and X-WZ were in charge of the data collection and analysis of this study. Y-HZ was responsible for all the procedures taken during data collection. S-LL was in charge of the formulation of the general research topic, the construction of the research framework, and the proposing of the theoretical hypothesis. All authors contributed to the article and approved the submitted version.

## Conflict of Interest

The authors declare that the research was conducted in the absence of any commercial or financial relationships that could be construed as a potential conflict of interest.

## Publisher’s Note

All claims expressed in this article are solely those of the authors and do not necessarily represent those of their affiliated organizations, or those of the publisher, the editors and the reviewers. Any product that may be evaluated in this article, or claim that may be made by its manufacturer, is not guaranteed or endorsed by the publisher.

## Footnotes

1
http://www.quantpsy.org/sobel/

2
http://www.afhayes.com
